# Genetically predicted circulating concentrations of micronutrients and risk of autoimmune thyroiditis: a Mendelian randomized study

**DOI:** 10.3389/fimmu.2024.1425351

**Published:** 2024-08-20

**Authors:** Rongliang Qiu, Xuemei Sha, Penghao Kuang, Fangsen Chen, Jinbo Fu

**Affiliations:** ^1^ The School of Clinical Medicine, Fujian Medical University, Fuzhou, China; ^2^ Department of General Surgery, Zhongshan Hospital, Xiamen University, Xiamen, China; ^3^ School of Medicine, Xiamen University, Xiamen, China

**Keywords:** autoimmune thyroiditis, micronutrients, copper, iron, calcium, vitamins, zinc

## Abstract

**Background:**

Micronutrients play pivotal roles in modulating various aspects of the immune response. However, the existing literature on the association between micronutrients and autoimmune thyroiditis (AIT) remains limited and contentious. To address this gap, we conducted Mendelian randomization (MR) to investigate potential links between genetically predicted concentrations of six micronutrients (Copper (Cu), Iron (Ir), Calcium (Ca), Vitamin D (VD), Vitamin C (VC), Zinc (Zn)) and the risk of AIT.

**Method:**

Utilizing summary statistics from genome-wide association studies (GWAS) in individuals of European descent, we employed MR methodologies to elucidate the interplay between micronutrients and AIT. Three distinct MR techniques were employed: Inverse Variance Weighted (IVW), MR-Egger regression, and Weighted Median Estimator (WME). Additionally, we evaluated outcome heterogeneity using Cochran’s Q statistic and assessed pleiotropy using the MR-Egger intercept.

**Result:**

IVW analysis revealed no substantial evidence supporting a significant impact of genetically predicted micronutrient concentrations on AIT risk (Cu: OR = 0.918, P = 0.875; Ir: OR = 0.653, P = 0.264; Ca: OR = 0.964, P = 0.906; VD: OR = 0.717, P = 0.378; VC: OR = 0.986, P = 0.875; Zn: OR = 0.789, P = 0.539). Cochran’s Q test for IVW indicated no notable heterogeneity. Moreover, the MR-Egger intercept method suggested the presence of horizontal pleiotropy between serum VC levels and AIT (MR-Egger intercept = −0.037, p = 0.026), while no such pleiotropy was observed for other micronutrients.

**Conclusion:**

Our MR analysis does not support a causal relationship between genetically predicted concentrations of six micronutrients (Cu, Ir, Ca, VD, VC, and Zn) and the risk of AIT.

## 1 Introduction

Autoimmune thyroiditis (AIT), also referred to as Hashimoto’s thyroiditis or chronic lymphocytic thyroiditis stands as one of the most prevalent organ-specific autoimmune disorders. It is typified by the presence of antibodies targeting thyroid antigens (thyroid peroxidase (TPO) and thyroglobulin (TG)), alongside diffuse lymphocytic infiltration of thyroid tissue. AIT exhibits an annual incidence ranging from 27 to 273 cases per 100,000 individuals (1, 2). The pathogenesis of AIT remains incompletely elucidated, though it is understood to be multifactorial, influenced by intricate interactions among multiple susceptibility genes and environmental factors such as stress, smoking, microbial infections, chemical pollutants, and dietary iodine (3).

The primary medical intervention for AIT-induced hypothyroidism involves daily oral administration of levothyroxine (LT4) to maintain normal thyroid-stimulating hormone (TSH) levels (4). Alongside LT4 therapy, dietary modifications and supplementation offer tangible benefits and constitute integral components of the therapeutic regimen. Observational controlled investigations have revealed frequent occurrences of micronutrient deficiencies in AIT patients (5). The deficiency or excess of any micronutrient (such as Copper (Cu), Iron (Ir), Zinc (Zn), iodine, and selenium) may affect the synthesis of TG and disrupt the homeostasis of the thyroid gland, thereby reducing the body’s immune ability and even disrupting the regulation of systemic inflammation (6, 7). Meanwhile, observational studies have also highlighted the important impact of various micronutrients, such as vitamin D (VD), antioxidants, monounsaturated and polyunsaturated fatty acids, magnesium, and Zn, as critical in reducing thyroid inflammation (8–10). Although current observational studies report associations between various micronutrients and AIT, concerns about potential bias from confounding factors cannot be completely eliminated. Furthermore, quantifying causal effects in traditional observational studies can be challenging due to residual confounding and reverse causation (11).

With the increase in statistical summary data from large-scale genome-wide association studies (GWAS), Mendelian randomization (MR) (12) has emerged as a potent tool. MR utilizes single-nucleotide polymorphisms (SNP) as instrumental variables (IV) strongly linked with exposure to probe causal effects between exposure and outcome, thereby enhancing the reliability of causal inferences and fostering robust etiological conclusions. Because genotypes are randomly assigned at conception, they provide an accurate representation of exposure that is not affected by potential confounders (such as environmental exposures) and does not change with the onset of disease. By leveraging genotypes randomly assigned at conception, MR circumvents potential confounding influences, such as environmental exposures, and remains unaffected by disease onset. Given the pivotal role of genetic susceptibility in AIT development, a genetic perspective is imperative. Accordingly, this study employs the two-sample MR approach to investigate potential causal links between genetically predicted circulating concentrations of micronutrients (Cu, Ir, Calcium (Ca), VD, Vitamin C (VC), Zn) and AIT.

## 2 Materials and methods

### 2.1 Study design

**Figure 1:**
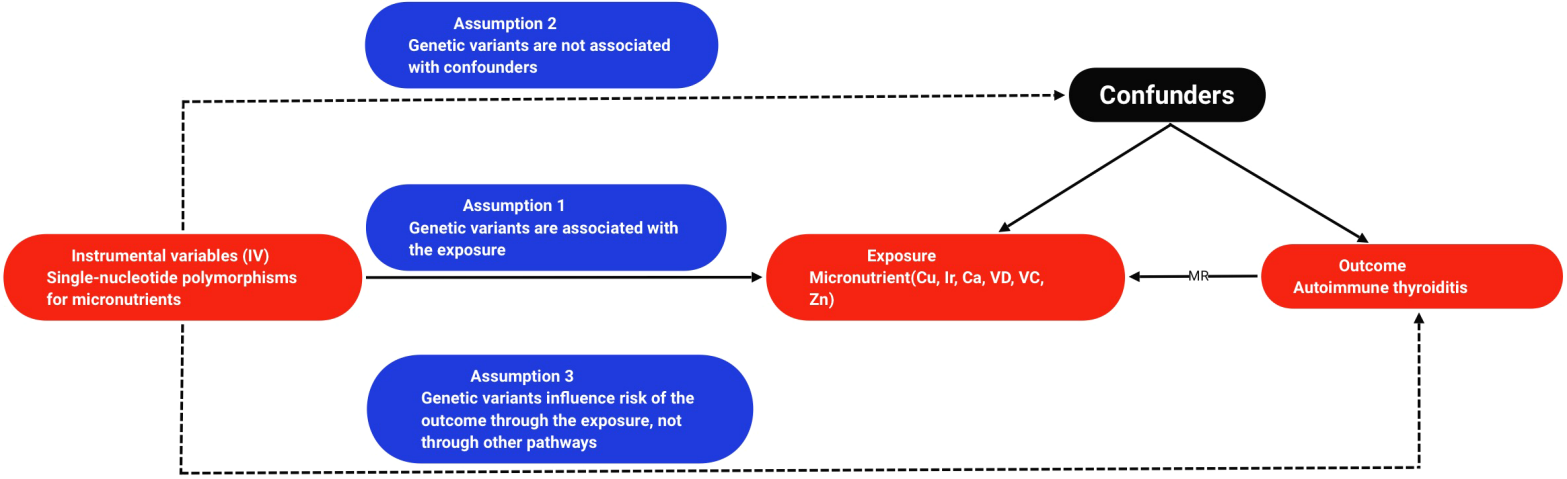
Flowchart of the design of a Mendelian randomized study of the causal association between micronutrients and autoimmune thyroiditis. Cu, copper; Ir, iron; Ca, calcium; VD, vitamin D; VC, vitamin C; Zn, zinc; MR, mendelian randomization.

This study employs a two-sample MR to examine the causal relationship between micronutrients and AIT, as depicted in **Figure 1** illustrating the research design. MR hinges on three fundamental assumptions: (1) the assumption of association, positing that genetic variation correlates with exposure; (2) the assumption of independence: genetic variation should not have any connections with confounding factors influencing the exposure-outcome relationship; and (3) the assumption of exclusivity, suggesting that genetic variation influences outcome solely through exposure pathways (13). All MR analyses utilized publicly available summary statistics, obviating the need for additional ethical approval or informed consent.

### 2.2 Data sources

In this study, we searched Open GWAS for statistical summary data related to micronutrient cycling concentrations as exposure (See **Table 1** for details). For Cu circulating concentrations, GWAS summary data encompassed 2,603 European individuals, revealing 2 SNP strongly associated with Cu levels (**Supplementary Table S1**). In the case of Ir circulating concentrations, the dataset comprised 23,986 individuals of European ancestry, identifying 3 significant SNP (**Supplementary Table S2**). Regarding Ca circulating concentrations, data encompassed 315,153 European individuals, identifying 212 significant SNP (**Supplementary Table S3**). VD circulating concentrations were assessed in 496,946 individuals of European descent, uncovering 118 significant SNP (**Supplementary Table S4**). VC circulating concentrations were examined in 291 European individuals, revealing 68 significant SNP (**Supplementary Table S5**). Finally, Zn circulating concentrations were investigated in 2,603 European individuals, identifying 2 significant SNP (**Supplementary Table S6**).

**Table 1:** Genetic summary data sources for micronutrients and autoimmune thyroiditis.

Trait	Sample size	Number of SNPs	Population	Sex	Year	PMID
Copper	2,603	2,543,646	European	Males and Females	2013	23720494
Iron	23,986	2,096,457	European	Males and Females	2014	25352340
Calcium	315,153	19,052,100	European	Males and Females	2021	34594039
Vitamin D	496,946	6,896,093	European	Males and Females	2020	32242144
Vitamin C	291	6,870,007	European	Males and Females	2021	33437055
Zinc	2,603	2,543,646	European	Males and Females	2013	23720494
Autoimmune thyroiditis	187,684	16,380,358	European	Males and Females	2021	NA

As for outcomes, GWAS summary statistics for AIT included 213,746 European individuals, comprising 244 AIT patients and 187,684 controls, sourced from the FinnGen database R8 (See **Table 1** for details).

### 2.3 Selection of genetic instrumental variable

The selection criteria for genetic IV were as follows: (1) Screening exposure databases to identify SNP loci of genome-wide significance (p < 5 × 10^-8^); (2) The linkage disequilibrium (LD) criterion set the R^2^ threshold (R^2^ < 0.001 and genetic distance = 10,000 kb), and only those SNPs exhibiting the most significant p-values were retained for subsequent analyses; and (3) Excluding SNP with F-statistics <10 (14) and palindromic sequences to ensure that the effects of SNP on exposure and outcome stemmed from the same allele.

### 2.4 Statistical analysis

In this investigation, we initially harmonized the alleles associated with both exposure (Cu, Ir, Ca, VD, VC, Zn) and outcome (AIT), following which a two-sample MR analysis was conducted. We employed three MR methodologies, namely Inverse Variance Weighted (IVW) (15), MR-Egger regression (16), and Weighted Median Estimator (WME) (17), to assess the causal relationship between micronutrients and AIT. The IVW method assumes the validity of all genetic variations. Through meta-analysis, it amalgamates the Wald estimate of each SNP and employs weighted linear regression to synthesize these estimates, thereby comprehensively evaluating the impact of micronutrients on AIT (18). IVW serves as the principal analytical tool, furnishing unbiased causal estimates in the absence of horizontal pleiotropy (19). WME and MR-Egger regression methods are utilized as supplementary methods to IVW estimation, as they can offer more reliable estimates under less stringent conditions.

To gauge the robustness and sensitivity of our findings, we conducted additional sensitivity analyses. Heterogeneity was assessed via Cochran’s Q test, computed using the IVW method. Furthermore, potential pleiotropy was evaluated and adjusted using the MR-Egger intercept test (20). Additionally, we scrutinized outliers that could potentially influence our MR estimates through the examination of forest plots, funnel plots, and scatter plots.

All the above-mentioned MR analyses in this study were performed using the “TwoSampleMR” package in R 4.1.1 software.

## 3 Results

### 3.1 Selection of genetic instrumental variable

Following a sequence of quality control procedures, a total of six genetic instruments were established for this study. These genetic instruments were constructed as follows: (1): For Cu exposure and AIT outcome, two SNP were included in the analysis after merging the relevant datasets. (2) For Ir exposure and AIT outcome, three SNP were included in the analysis following the merging of datasets. (3) Upon merging Ca exposure and AIT outcome datasets, and subsequent removal of 8 palindromic sequences (rs12626330, rs1763519, rs296849, rs4517550, rs4744854, rs490275, rs72941253, rs7839633), a total of 195 SNP were retained for analysis. (4) After merging VD exposure and AIT outcome datasets, and excluding 2 palindromic sequences (rs57601828, rs7955128), 115 SNP were ultimately included in the analysis. (5) The integration of VC exposure and AIT outcome datasets resulted in the inclusion of 65 SNPs for analysis. (6) Lastly, the combination of Zn exposure and AIT outcome datasets included two SNPs for analysis.

### 3.2 Mendelian randomization analysis

We conducted a two-sample MR study on genetically predicted micronutrient circulating concentrations and AIT, yielding no compelling evidence to support a significant causal relationship between genetically predicted micronutrient concentrations and AIT (refer to **Table 2** for detailed outcomes).

1. Cu: IVW analysis (OR = 0.918, 95% CI = 0.315-2.674; P = 0.875) revealed no discernible causal relationship between serum Cu concentration and AIT.
2. Ir: IVW analysis (OR = 0.653, 95% CI = 0.309-1.379; P = 0.264) demonstrated no substantial evidence supporting a causal relationship between serum Ir concentration and AIT. Consistent causal effects were observed in analyses employing WME (OR = 0.639, 95% CI = 0.288-1.415; P = 0.269) and MR-Egger regression (OR = 1.009, 95% CI = 0.218-4.676; P = 0.993) methods.
3. Ca: The results of the IVW (OR = 0.964, 95% CI = 0.523-1.776; P = 0.906), WME (OR = 1.342, 95% CI = 0.435-4.139; P = 0.609), and MR-Egger regression (OR = 1.310, 95% CI = 0.436-3.935; *P* = 0.631) analyses collectively failed to provide evidence supporting a causal link between serum Ca concentrations and AIT.
4. VD: The results of IVW (OR = 0.717, 95% CI = 0.341-1.504; P = 0.378), WME (OR = 1.265, 95% CI = 0.398-4.026; P = 0.690) and MR-Egger regression (OR = 1.951, 95% CI = 0.623-6.111; *P* = 0.254) method analyses indicated no observable causal relationship between serum VD concentration and AIT.
5. VC: IVW analyses (OR = 0.986, 95% CI = 0.828-1.175; P = 0.875) provided no evidence supporting a causal relationship between serum VC concentrations and AIT. Comparable causal effects were observed in WME (OR = 0.845, 95% CI = 0.645-1.108; P = 0.223) and MR-Egger regression (OR = 0.847, 95% CI = 0.549-1.306; P = 0.455) analyses.
6. Zn: IVW analysis (OR = 0.789, 95% CI = 0.370-1.682; P = 0.539) yielded no indication of a causal relationship between serum Zn concentration and AIT.

**Table 2:** Mendelian randomized estimation of the association between micronutrients and autoimmune thyroiditis.

Exposure	Outcome	IVW				MR-Egger				Weighted median	
		OR (95% CI)	P-value	Cochran Q	P-value	OR (95% CI)	P-value	MR-Egger intercept	P-value	OR (95% CI)	P-value
Copper	Autoimmune thyroiditis	0.918 (0.315-2.674)	0.875	3.069	0.080	NA	NA	NA	NA	NA	NA
Iron	Autoimmune thyroiditis	0.653 (0.309-1.379)	0.264	0.756	0.683	1.009 (0.218-4.676)	0.993	-0.086	0.639	1.639 (0.288-1.415)	0.269
Calcium	Autoimmune thyroiditis	0.964 (0.523-1.776)	0.906	216.124	0.132	1.310 (0.436-3.935)	0.631	-0.010	0.512	1.342 (0.435-4.139)	0.609
Vitamin D	Autoimmune thyroiditis	0.717 (0.341-1.504)	0.378	90.420	0.949	1.951 (0.623-6.111)	0.254	-0.037	0.026	1.265 (0.398-4.026)	0.690
Vitamin C	Autoimmune thyroiditis	0.986 (0.828-1.175)	0.875	76.166	0.142	0.847 (0.549-1.306)	0.455	0.089	0.454	0.845 (0.645-1.108)	0.223
Zinc	Autoimmune thyroiditis	0.789 (0.370-1.682)	0.539	1.479	0.224	NA	NA	NA	NA	NA	NA

NA, Insufficient number of SNPs for analysis.

### 3.3 Sensitivity analysis

In our sensitivity analysis, we initially employed IVW’s Cochran’s Q test to assess the heterogeneity of results. The findings revealed that all analyses yielded p-values exceeding 0.05, indicating the absence of significant heterogeneity in our study (refer to **Table 2**). Subsequently, we utilized the MR-Egger intercept method to scrutinize horizontal pleiotropy. Results indicated that the p-value of the MR-Egger intercept method for serum VC and AIT was below 0.05 (MR-Egger intercept = -0.037, p = 0.026), suggestive of the presence of horizontal pleiotropy. Conversely, p-values of the MR-Egger intercept method for other scenarios surpassed 0.05, signifying the absence of horizontal pleiotropy (refer to **Table 2**). Additionally, we conducted visual inspections of the funnel plot, which displays a roughly symmetrical distribution, implying a relatively low risk of bias and high reliability of the results. Further scrutiny involved the examination of scatter plots, wherein each point represented an instrumental variable. The forest plot depicted each horizontal solid line reflecting the outcome of a single SNP estimated using the Wald ratio method. Detailed scatter plots, funnel plots, and forest plots are available in the **Supplementary Material**.

## 4 Discussions

In this two-sample MR analysis investigating the relationship between six micronutrient concentrations and the risk of AIT, we did not identify a significant association between genetically predicted circulating micronutrient concentrations and the risk of AIT.

Cu functions by binding to ceruloplasmin in plasma, thereby stimulating the activities of both innate and adaptive immunity. A population-based study utilizing data from the 2011-2012 U.S. National Health and Nutrition Examination Survey(NHANES) revealed that serum Cu concentrations were 20% higher in women compared to men. Furthermore, it was observed that circulating serum Cu concentrations correlated with elevated levels of free thyroxine (fT4) and total thyroxine (tT4) in women, whereas in men, circulating Cu concentrations correlated with elevated levels of total triiodothyronine (tT3) and tT4 (21). Another study indicated that Cu proportion may directly impact thyroid function in individuals with AIT or overt hypothyroidism (22). Moreover, Cu may influence thyroid hormone levels through autoimmune mechanisms, given the close association between thyroid autoimmunity and dysfunction (23). While observational studies suggest an association between Cu and AIT, our study reveals no relationship between genetically predicted circulating Cu concentrations and AIT.

Ir, found in plasma as a constituent of hemoglobin, myoglobin, enzymes, and other proteins, plays a regulatory role in innate immunity by modulating monocytes and neutrophils. TPO is a heme enzyme that becomes active only upon binding to heme—a non-protein pseudogroup containing Fe^2+^ ions—and is primarily responsible for synthesizing thyroid hormones. Consequently, Ir deficiency impairs thyroid metabolic function. Of note, low Ir stores may contribute to the persistence of symptoms after LT4 therapy in 5%-10% of patients with hypothyroidism (24). Patients with AIT often suffer from autoimmune gastritis, which leads to reduced Fe absorption, or celiac disease, which causes Fe loss, and then develops Fe deficiency (25). Findings from a retrospective study involving 180 female patients with positive thyroid autoantibodies revealed higher frequencies of abnormal hemoglobin, Ir, and ferritin levels compared to healthy controls. Additionally, a negative correlation was observed between Thyroid Peroxidase Antibodies (TPOAb) levels and serum ferritin and Ir levels (26). Another cross-sectional study involving 7463 pregnant women and 2185 nonpregnant women with subclinical hypothyroidism demonstrated an association between Ir deficiency and a higher prevalence of isolated TPOAb positivity among pregnant women and nonpregnant women of childbearing age (27). Despite observational studies suggesting an association between Ir and AIT, our investigation reveals no causal relationship between genetically predicted circulating concentrations of Ir and AIT.

Thyroid disease significantly influences mineral metabolism, particularly the mineral density of bone tissue. This is attributed to the involvement of thyroid hormone in regulating the metabolism of Ca and phosphorus, with Ca being an essential mineral in bone tissue. In patients with hypothyroidism, the mean serum Ca concentration tends to decrease, and there exists a negative correlation between Ca and TSH (28, 29). Conversely, patients with hyperthyroidism often exhibit elevated serum Ca levels, potentially increasing the risk of osteoporosis and fractures (30). While observational studies have not directly established a link between Ca and AIT, our investigation reveals no causal relationship between genetically predicted circulating Ca concentrations and AIT.

VD is primarily synthesized endogenously following exposure to sunlight, particularly UVB radiation, through the skin. It plays a crucial role in regulating calcium-phosphate metabolism and promoting bone homeostasis. Recent research has unveiled VD’s immunomodulatory functions within both the innate and adaptive immune systems, suggesting its potential to foster immune tolerance, which, in turn, could inhibit the immunopathological processes underlying AIT (8). A study conducted among Polish women revealed an inverse correlation between TSH, TPOAb, thyroglobulin antibodies (TGAb), and serum VD levels across healthy individuals, AIT patients, and those with hypothyroidism (31). Similarly, another study demonstrated significantly lower serum VD levels in AIT patients compared to controls, with the severity of VD deficiency correlating with the duration of AIT and thyroid antibody levels (32). While some observational studies have suggested a potential association between VD deficiency and an elevated risk of AIT (33–35), others have failed to establish a clear link between serum VD levels and antithyroid antibodies or thyroid function (36, 37). These inconsistencies may stem from various factors, yet our investigation did not uncover a causal relationship between circulating VD concentration and AIT. Thus, further randomized, double-blind, placebo-controlled trials are warranted to elucidate the ambiguous causal relationship between VD and thyroid disease.

VC not only safeguards thyroid acini from oxidative damage owing to its antioxidant properties but may also potentially aid in the restoration of thyroid function through its non-oxidative activity, thereby facilitating the recovery of thyroid hormone synthesis function (38). For example, VC has been shown to contribute to the synthesis of paraoxonase, which contributes to the detoxification of OPs (39). While investigations into the role of VC in thyroid disease are limited, our study marks the first exploration of the causal relationship between VC and AIT. Nevertheless, studies have revealed no causal effect between circulating VC concentration and AIT.

Zn plays a key role in thyroid hormone metabolism by regulating deiodinase activity, the synthesis of thyrotropin-releasing hormone (TRH) and TSH enzymes, and the structure of transcription factors essential for thyroid hormone synthesis (40). Numerous studies in the literature have explored the relationship between Zn and thyroid hormones, with both hypothyroidism and hyperthyroidism being linked to low Zn concentrations (41). Zn deficiency can disrupt thyroid hormone levels and potentially lead to elevated antibody titers against thyroid antigens (42). Moreover, autoimmune diseases are associated with pathological alterations in circulating Zn concentrations (43). However, our study did not uncover a causal association between circulating Zn concentrations and AIT.

Previous research on the relationship between micronutrients and AIT was controversial. This study marks the first attempt to investigate the causal connection between micronutrients and AIT utilizing a two-sample MR approach. Importantly, employing distinct databases for exposure and outcome datasets helped mitigate the risk of bias arising from overlapping samples. Furthermore, the selection of SNP with robust correlations (p < 5×10−8) and high intensity (F statistic > 10) to construct IV bolstered the comparability and credibility of the study. Nevertheless, it is imperative to acknowledge the study’s limitations. Firstly, in the two-sample MR analyses of exposure (Cu, Ir, and Zn) and outcome (AIT), the inclusion of a small number of SNP—2, 3, and 2, respectively—unable the testing for pleiotropic effects. Secondly, the study sample comprised individuals of European ancestry, potentially constraining the generalizability of our findings to other populations.

## 5 Conclusion

In conclusion, our study findings indicate the absence of a causal relationship between genetically predicted circulating concentrations of six micronutrients (Cu, Ir, Ca, VD, VC, Zn) and the risk of AIT. Future studies are warranted to elucidate the effects of micronutrients on AIT and unravel their underlying mechanisms.

## Data Availability

The original contributions presented in the study are included in the article/**Supplementary Material**. Further inquiries can be directed to the corresponding author.
