# Supplementary material for: Genetically predicted circulating concentrations of micronutrients and risk of autoimmune thyroiditis: a Mendelian randomized study
Source: Front Immunol. 2024 Aug 20;15:1425351. doi: 10.3389/fimmu.2024.1425351 (PMC11368795; doi:10.3389/fimmu.2024.1425351)
Supplement: Supplementary file 1 [file DataSheet1.docx]

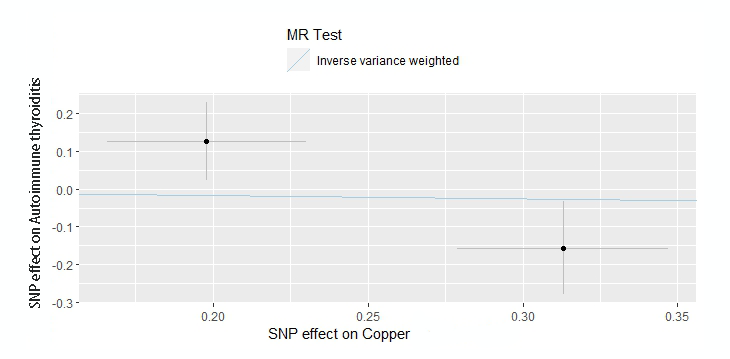
Figure S1: Scatter plot of the causal effect of copper on autoimmune thyroiditis, with the slope of each line corresponding to estimated causal effect per method.


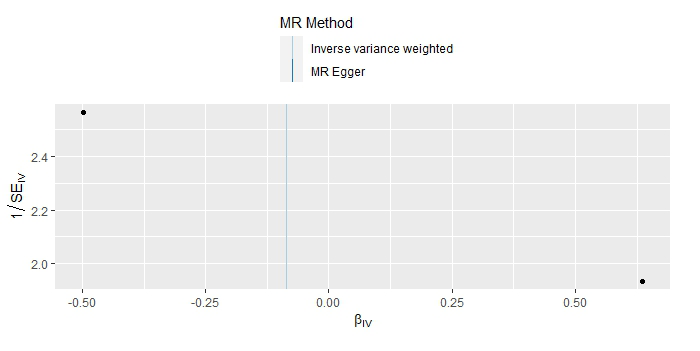
Figure S2: Funnel plot of the causal effect of copper on autoimmune thyroiditis.


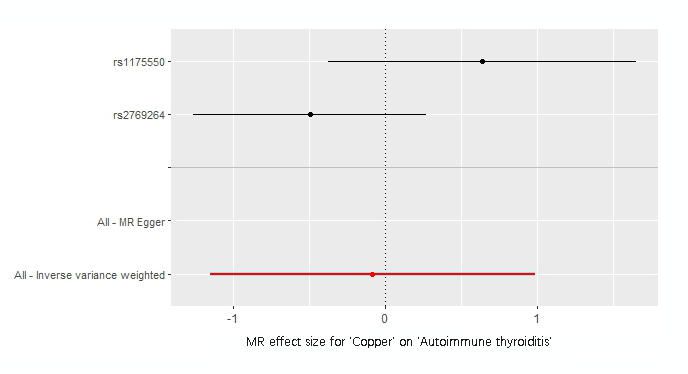
Figure S3: Forest plots of the causal effect of copper on autoimmune thyroiditis.


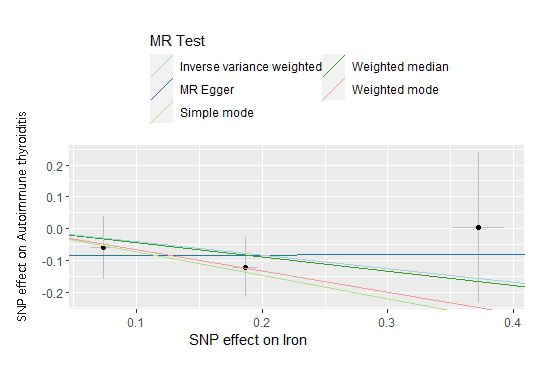
Figure S4: Scatter plot of the causal effect of iron on autoimmune thyroiditis, with the slope of each line corresponding to estimated causal effect per method.


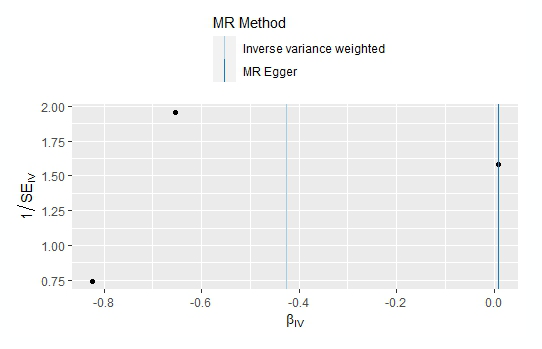
Figure S5: Funnel plot of the causal effect of iron on autoimmune thyroiditis.


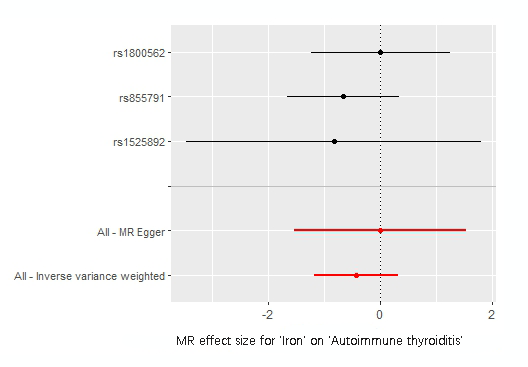


Figure S6: Forest plots of the causal effect of iron on autoimmune thyroiditis.


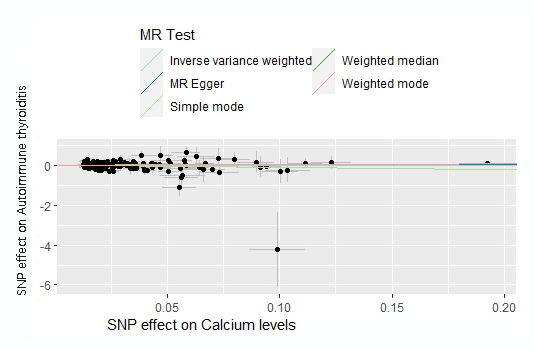


Figure S7: Scatter plot of the causal effect of calcium on autoimmune thyroiditis, with the slope of each line corresponding to estimated causal effect per method.


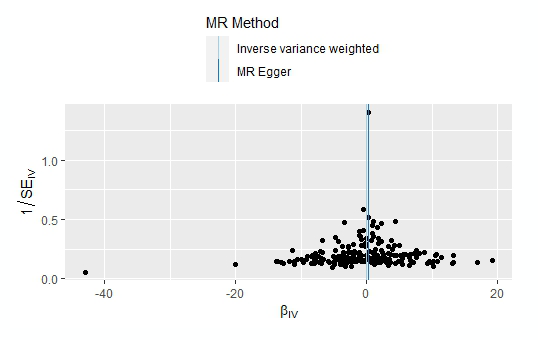
 Figure S8: Funnel plot of the causal effect of calcium on autoimmune thyroiditis.


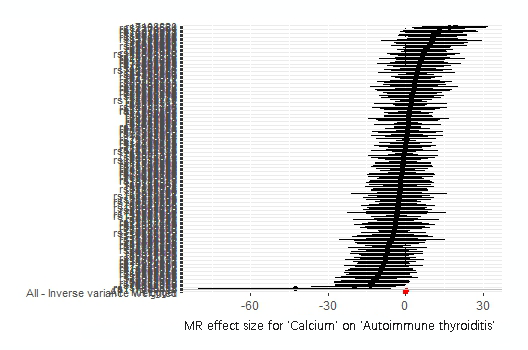


Figure S9: Forest plots of the causal effect of calcium on autoimmune thyroiditis.


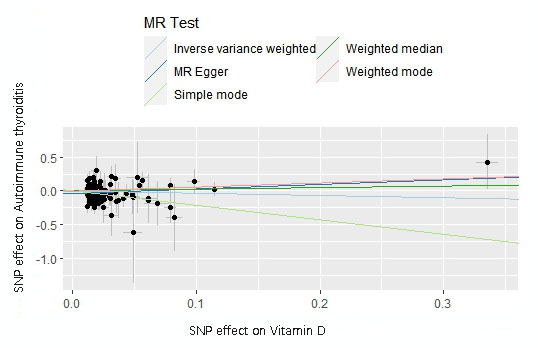


Figure S10: Scatter plot of the causal effect of vitamin D on autoimmune thyroiditis, with the slope of each line corresponding to estimated causal effect per method.


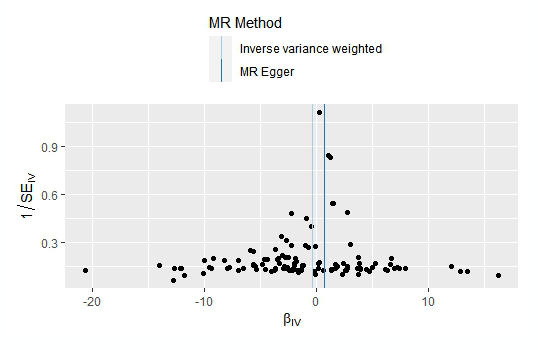


Figure S11: Funnel plot of the causal effect of vitamin D on autoimmune thyroiditis.


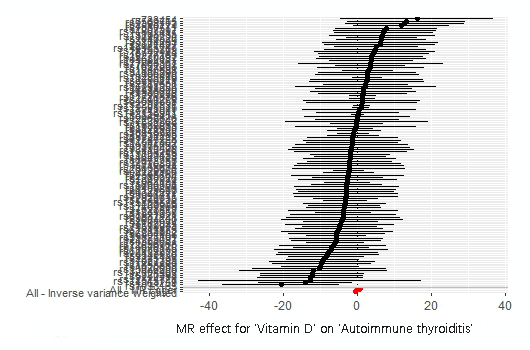


Figure S12: Forest plots of the causal effect of vitamin D on autoimmune thyroiditis.


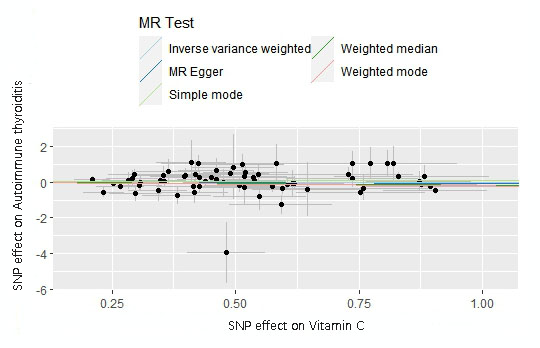


Figure S13: Scatter plot of the causal effect of vitamin C on autoimmune thyroiditis, with the slope of each line corresponding to estimated causal effect per method.


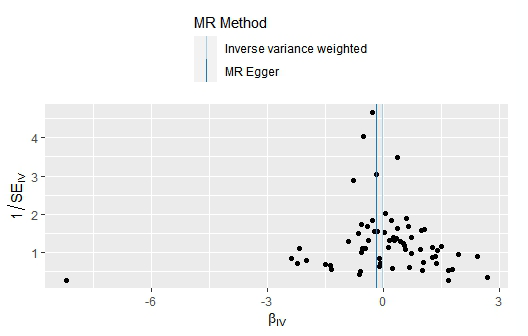


Figure S14: Funnel plot of the causal effect of vitamin C on autoimmune thyroiditis.


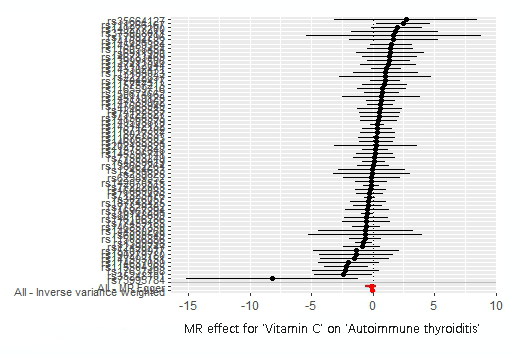


Figure S15: Forest plots of the causal effect of vitamin C on autoimmune thyroiditis.


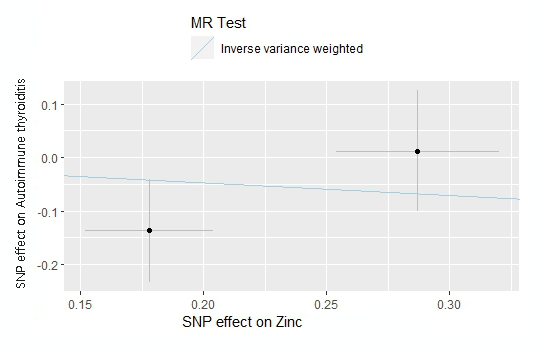


Figure S16: Scatter plot of the causal effect of zinc on autoimmune thyroiditis, with the slope of each line corresponding to estimated causal effect per method.


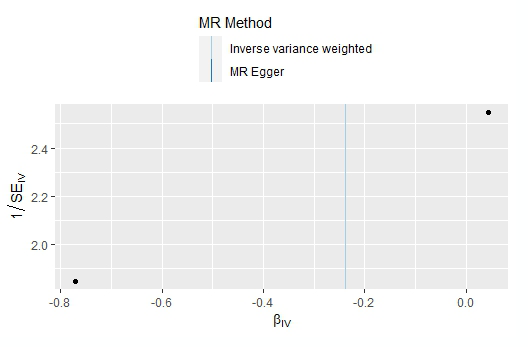


Figure S17: Funnel plot of the causal effect of zinc on autoimmune thyroiditis.


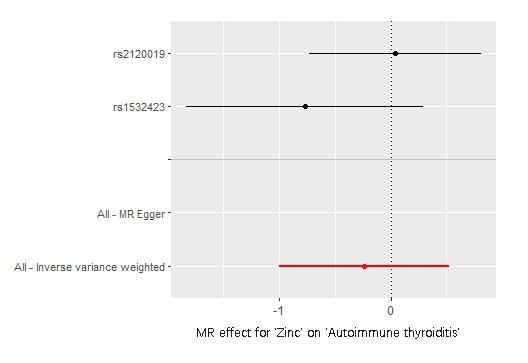


Figure S18: Forest plots of the causal effect of zinc on autoimmune thyroiditis.

Supplementary Table 1. 2 SNPs associated with Copper.

| SNP | Chromosome | position | Beta | Se | Effect allele | P | F |
| --- | --- | --- | --- | --- | --- | --- | --- |
| rs1175550 | 1 | 3691528 | 0.198 | 0.032 | G | 5.03002E-10 | 38.28515625 |
| rs2769264 | 1 | 151344741 | 0.313 | 0.034 | G | 2.63027E-20 | 84.7482699 |

Supplementary Table 2. 3 SNPs associated with Iron.

| SNP | Chromosome | position | Beta | Se | Effect allele | P | F |
| --- | --- | --- | --- | --- | --- | --- | --- |
| rs1525892 | 3 | 133484712 | 0.0736 | 0.0104 | A | 1.65082E-12 | 50.08284024 |
| rs1800562 | 6 | 26093141 | 0.3724 | 0.02 | A | 3.95913E-77 | 346.7044 |
| rs855791 | 22 | 37462936 | 0.1868 | 0.0101 | G | 4.31221E-77 | 342.0668562 |

Supplementary Table 3. 212 SNPs associated with Calcium.

| SNP | Chromosome | position | Beta | Se | Effect allele | P | F |
| --- | --- | --- | --- | --- | --- | --- | --- |
| rs75895430 | 1 | 68268784 | 0.0796 | 0.0071 | G | 5.31E-29 | 125.6925213 |
| rs71658797 | 1 | 77967507 | 0.0211 | 0.0038 | A | 2.36E-08 | 30.83171745 |
| rs9428344 | 1 | 116816699 | 0.0124 | 0.0022 | T | 2.22E-08 | 31.76859504 |
| rs12135382 | 1 | 1098421 | 0.0222 | 0.0026 | T | 1.29E-17 | 72.90532544 |
| rs12132412 | 1 | 21820042 | 0.0276 | 0.0024 | G | 8.06E-31 | 132.25 |
| rs12411216 | 1 | 155164480 | 0.0254 | 0.0023 | A | 3.87E-28 | 121.9584121 |
| rs10917386 | 1 | 23799001 | 0.0192 | 0.0023 | T | 2.36E-16 | 69.68620038 |
| rs11204766 | 1 | 151018060 | 0.0411 | 0.0045 | C | 1.30E-19 | 83.41777778 |
| rs7546838 | 1 | 156785771 | 0.017 | 0.0024 | G | 1.67E-12 | 50.17361111 |
| rs11588907 | 1 | 178509462 | -0.0159 | 0.0026 | T | 1.19E-09 | 37.39792899 |
| rs1434282 | 1 | 199010721 | 0.0196 | 0.0025 | T | 8.03E-15 | 61.4656 |
| rs116631899 | 1 | 52787887 | 0.0732 | 0.0088 | C | 8.00E-17 | 69.19214876 |
| rs697852 | 1 | 226914734 | -0.0207 | 0.0032 | A | 1.65E-10 | 41.84472656 |
| rs924204 | 1 | 16513926 | 0.0195 | 0.0023 | G | 7.10E-17 | 71.88090737 |
| rs841572 | 1 | 43436051 | 0.0297 | 0.0022 | A | 1.52E-40 | 182.25 |
| rs1497826 | 1 | 217471419 | 0.0229 | 0.0024 | G | 3.66E-21 | 91.04340278 |
| rs10863512 | 1 | 220075215 | -0.0199 | 0.0023 | T | 2.72E-18 | 74.86011342 |
| rs116769926 | 2 | 9007708 | 0.0726 | 0.0082 | A | 6.58E-19 | 78.38726948 |
| rs12998379 | 2 | 10163922 | -0.0219 | 0.0029 | A | 7.17E-14 | 57.02853746 |
| rs76170039 | 2 | 54846795 | 0.0203 | 0.0031 | TAA | 5.78E-11 | 42.88137357 |
| rs35751693 | 2 | 98242555 | 0.0433 | 0.0066 | T | 5.15E-11 | 43.04155188 |
| rs778368 | 2 | 233745333 | -0.0128 | 0.0023 | C | 1.70E-08 | 30.97164461 |
| rs838717 | 2 | 234296444 | -0.0431 | 0.0022 | A | 4.42E-84 | 383.803719 |
| rs74847504 | 2 | 69639612 | 0.016 | 0.0022 | G | 5.38E-13 | 52.89256198 |
| rs1667305 | 2 | 232403829 | 0.0125 | 0.0023 | C | 3.06E-08 | 29.536862 |
| rs10182990 | 2 | 242403940 | -0.0205 | 0.0026 | G | 3.36E-15 | 62.16715976 |
| rs35095338 | 2 | 191357694 | 0.0147 | 0.0022 | T | 3.29E-11 | 44.64669421 |
| rs7559013 | 2 | 213735555 | 0.0253 | 0.0037 | C | 7.55E-12 | 46.7560263 |
| rs2971855 | 2 | 234373537 | 0.0172 | 0.0025 | A | 8.01E-12 | 47.3344 |
| rs1260326 | 2 | 27730940 | -0.0464 | 0.0022 | C | 1.25E-95 | 444.8264463 |
| rs1374161 | 2 | 114034627 | -0.0206 | 0.0023 | A | 8.89E-20 | 80.21928166 |
| rs3748861 | 2 | 133196452 | -0.0169 | 0.0028 | A | 2.44E-09 | 36.42984694 |
| rs13389219 | 2 | 165528876 | -0.0162 | 0.0024 | T | 2.68E-11 | 45.5625 |
| rs146582485 | 2 | 10122470 | -0.021 | 0.0031 | CGCAGTGGTTT | 1.96E-11 | 45.88969823 |
| rs7587636 | 2 | 25518862 | -0.015 | 0.0022 | A | 2.47E-11 | 46.48760331 |
| rs4430924 | 2 | 61703856 | -0.028 | 0.0025 | G | 2.90E-28 | 125.44 |
| rs72941253 | 2 | 97392707 | -0.0382 | 0.0025 | G | 3.12E-51 | 233.4784 |
| rs6542581 | 2 | 121017318 | -0.022 | 0.0023 | G | 7.45E-21 | 91.49338374 |
| rs7592216 | 2 | 182743246 | -0.0206 | 0.0036 | T | 8.65E-09 | 32.74382716 |
| rs715 | 2 | 211543055 | -0.0138 | 0.0025 | C | 3.07E-08 | 30.4704 |
| rs147836956 | 3 | 122136197 | -0.0626 | 0.0085 | ACT | 1.48E-13 | 54.23889273 |
| rs4685773 | 3 | 4632637 | 0.0293 | 0.0041 | T | 8.65E-13 | 51.07019631 |
| rs1899951 | 3 | 12394840 | -0.0346 | 0.0036 | T | 2.58E-21 | 92.37345679 |
| rs10513810 | 3 | 186667885 | -0.0268 | 0.0039 | G | 4.19E-12 | 47.22156476 |
| rs648514 | 3 | 52467263 | -0.0144 | 0.0022 | A | 1.03E-10 | 42.84297521 |
| rs1354034 | 3 | 56849749 | -0.016 | 0.0022 | C | 7.29E-13 | 52.89256198 |
| rs141079827 | 3 | 121456218 | -0.0555 | 0.0091 | A | 1.12E-09 | 37.19659461 |
| rs34395935 | 3 | 121937961 | 0.0576 | 0.0033 | C | 9.92E-67 | 304.661157 |
| rs73186030 | 3 | 122013465 | 0.1927 | 0.0036 | T | 1.00E-200 | 2865.222994 |
| rs76249487 | 3 | 122036904 | -0.0395 | 0.0048 | A | 1.17E-16 | 67.71918403 |
| rs140559425 | 3 | 156512415 | -0.0149 | 0.0022 | AAAT | 3.35E-11 | 45.86983471 |
| rs10049088 | 3 | 156797648 | 0.0139 | 0.0022 | T | 6.86E-10 | 39.91942149 |
| rs138789759 | 3 | 107190555 | 0.0469 | 0.0047 | A | 3.47E-23 | 99.57492078 |
| rs73186098 | 3 | 122092504 | -0.0914 | 0.0106 | C | 8.41E-18 | 74.3499466 |
| rs3905668 | 3 | 135931586 | 0.0258 | 0.0027 | G | 6.86E-21 | 91.30864198 |
| rs9811323 | 3 | 177507406 | 0.0148 | 0.0026 | C | 1.87E-08 | 32.40236686 |
| rs13073106 | 3 | 186342060 | 0.0341 | 0.0023 | T | 2.41E-48 | 219.8128544 |
| rs6841258 | 4 | 40565426 | -0.0361 | 0.0029 | T | 1.51E-34 | 154.9595719 |
| rs183515408 | 4 | 106214477 | 0.0226 | 0.0037 | T | 1.17E-09 | 37.30898466 |
| rs77849807 | 4 | 26152727 | 0.0566 | 0.01 | G | 1.36E-08 | 32.0356 |
| rs72660383 | 4 | 75949596 | -0.0261 | 0.0046 | C | 1.58E-08 | 32.19328922 |
| rs11730491 | 4 | 26174563 | 0.0169 | 0.0029 | T | 4.34E-09 | 33.960761 |
| rs7655631 | 4 | 26869338 | -0.0148 | 0.0024 | C | 7.12E-10 | 38.02777778 |
| rs7688574 | 4 | 38533499 | 0.0139 | 0.0025 | T | 3.30E-08 | 30.9136 |
| rs62309863 | 4 | 115390971 | -0.0159 | 0.0022 | T | 1.11E-12 | 52.23347107 |
| rs13108218 | 4 | 3443931 | -0.0393 | 0.0023 | G | 2.22E-67 | 291.9640832 |
| rs13107325 | 4 | 103188709 | -0.064 | 0.0047 | T | 5.42E-42 | 185.4232684 |
| rs4320103 | 4 | 170910322 | 0.0348 | 0.0057 | G | 8.52E-10 | 37.27423823 |
| rs62362193 | 5 | 72345509 | 0.0163 | 0.0023 | A | 2.84E-12 | 50.22495274 |
| rs4976647 | 5 | 176788622 | 0.02 | 0.0023 | C | 7.35E-18 | 75.61436673 |
| rs35096828 | 5 | 133897439 | -0.0233 | 0.0026 | C | 1.16E-18 | 80.3091716 |
| rs11743466 | 5 | 174045907 | 0.0145 | 0.0023 | G | 3.88E-10 | 39.74480151 |
| rs10942734 | 5 | 74595194 | 0.015 | 0.0022 | C | 1.44E-11 | 46.48760331 |
| rs657075 | 5 | 131430118 | -0.0177 | 0.0032 | A | 3.91E-08 | 30.59472656 |
| rs9390702 | 6 | 101347446 | 0.0129 | 0.0022 | T | 7.65E-09 | 34.3822314 |
| rs9401792 | 6 | 125324865 | -0.0216 | 0.0023 | G | 4.57E-20 | 88.19659735 |
| rs945890 | 6 | 130321899 | -0.0149 | 0.0024 | T | 1.06E-09 | 38.54340278 |
| rs1763519 | 6 | 134518919 | -0.0295 | 0.0023 | C | 1.54E-36 | 164.5085066 |
| rs490275 | 6 | 160765831 | -0.0134 | 0.0022 | T | 2.46E-09 | 37.09917355 |
| rs6597256 | 6 | 7208007 | -0.0127 | 0.0023 | A | 2.06E-08 | 30.48960302 |
| rs9379881 | 6 | 10877867 | -0.0136 | 0.0022 | C | 9.96E-10 | 38.21487603 |
| rs915896 | 6 | 32187721 | -0.0168 | 0.0023 | T | 7.15E-13 | 53.35349716 |
| rs2327774 | 6 | 137275839 | -0.0199 | 0.0023 | C | 1.15E-17 | 74.86011342 |
| rs1933737 | 6 | 116310287 | -0.0147 | 0.0025 | C | 8.21E-09 | 34.5744 |
| rs1187115 | 6 | 34172055 | -0.0425 | 0.0033 | A | 6.14E-38 | 165.8631772 |
| rs9356996 | 6 | 26008487 | -0.0166 | 0.0024 | A | 5.27E-12 | 47.84027778 |
| rs7769064 | 6 | 74495849 | -0.0393 | 0.0023 | G | 5.08E-63 | 291.9640832 |
| rs212837 | 7 | 26695215 | 0.0127 | 0.0023 | C | 2.89E-08 | 30.48960302 |
| rs17164683 | 7 | 92286980 | -0.0216 | 0.0028 | T | 7.32E-15 | 59.51020408 |
| rs11772303 | 7 | 127398962 | 0.013 | 0.0024 | T | 3.71E-08 | 29.34027778 |
| rs4718271 | 7 | 65212402 | 0.0307 | 0.0025 | C | 6.87E-35 | 150.7984 |
| rs7786368 | 7 | 77500734 | -0.0262 | 0.0023 | C | 4.94E-30 | 129.7618147 |
| rs62483619 | 7 | 106796537 | -0.0215 | 0.0025 | T | 2.28E-17 | 73.96 |
| rs3857708 | 7 | 16140077 | 0.0192 | 0.0025 | A | 3.57E-14 | 58.9824 |
| rs114949263 | 7 | 150498245 | 0.034 | 0.0039 | C | 7.86E-18 | 76.00262985 |
| rs41393948 | 8 | 8296230 | -0.018 | 0.0031 | T | 1.10E-08 | 33.71488033 |
| rs4841132 | 8 | 9183596 | 0.0583 | 0.0042 | G | 4.68E-43 | 192.680839 |
| rs4647903 | 8 | 38272582 | 0.019 | 0.0025 | A | 6.67E-14 | 57.76 |
| rs2309233 | 8 | 21945518 | 0.0213 | 0.0028 | C | 5.91E-14 | 57.86862245 |
| rs36104352 | 8 | 23377604 | 0.0239 | 0.0034 | C | 1.89E-12 | 49.41262976 |
| rs2343592 | 8 | 106572270 | -0.0216 | 0.0024 | G | 7.71E-19 | 81 |
| rs12334564 | 8 | 124780824 | -0.0136 | 0.0024 | A | 1.29E-08 | 32.11111111 |
| rs2016749 | 8 | 143836118 | -0.0143 | 0.0025 | A | 1.78E-08 | 32.7184 |
| rs7003580 | 8 | 145007534 | 0.015 | 0.0023 | T | 1.19E-10 | 42.53308129 |
| rs16930077 | 8 | 63920229 | -0.0169 | 0.0026 | A | 6.57E-11 | 42.25 |
| rs7839633 | 8 | 98812248 | -0.0123 | 0.0022 | G | 2.75E-08 | 31.25826446 |
| rs3133548 | 8 | 101733229 | 0.0163 | 0.0029 | T | 2.33E-08 | 31.5921522 |
| rs12378991 | 9 | 77472066 | -0.0382 | 0.0046 | A | 9.51E-17 | 68.96219282 |
| rs12337706 | 9 | 97523154 | -0.0514 | 0.0042 | G | 1.88E-34 | 149.7709751 |
| rs550057 | 9 | 136146597 | -0.0142 | 0.0025 | T | 1.58E-08 | 32.2624 |
| rs296849 | 9 | 4780007 | 0.0123 | 0.0022 | A | 3.27E-08 | 31.25826446 |
| rs7856502 | 9 | 904078 | -0.0196 | 0.0026 | T | 5.22E-14 | 56.82840237 |
| rs883951 | 9 | 71514547 | 0.0233 | 0.0026 | G | 1.02E-18 | 80.3091716 |
| rs7864156 | 9 | 96905219 | 0.0191 | 0.0025 | G | 6.61E-14 | 58.3696 |
| rs4978466 | 9 | 114599048 | -0.0143 | 0.0026 | A | 3.02E-08 | 30.25 |
| rs4744854 | 9 | 80498559 | -0.0283 | 0.0023 | C | 2.48E-33 | 151.3969754 |
| rs518636 | 9 | 116363146 | 0.0138 | 0.0025 | G | 3.24E-08 | 30.4704 |
| rs10739679 | 9 | 129294471 | 0.0325 | 0.0023 | G | 3.33E-45 | 199.6691871 |
| rs498490 | 10 | 8118677 | -0.0261 | 0.0028 | T | 3.27E-20 | 86.88903061 |
| rs112371897 | 10 | 9321880 | 0.0698 | 0.0042 | T | 1.06E-60 | 276.1927438 |
| rs12416595 | 10 | 22409964 | 0.0152 | 0.0027 | G | 2.25E-08 | 31.69272977 |
| rs17774672 | 10 | 50507709 | -0.0288 | 0.0032 | A | 1.16E-19 | 81 |
| rs9415676 | 10 | 65010626 | 0.0147 | 0.0023 | G | 2.59E-10 | 40.84877127 |
| rs5786388 | 10 | 80999929 | 0.0188 | 0.0022 | CA | 3.93E-17 | 73.02479339 |
| rs1061134 | 10 | 100189252 | -0.0216 | 0.0038 | A | 8.71E-09 | 32.31024931 |
| rs7086226 | 10 | 22459979 | 0.0191 | 0.0026 | G | 5.31E-13 | 53.96597633 |
| rs4082330 | 10 | 65497266 | 0.0208 | 0.0032 | T | 1.12E-10 | 42.25 |
| rs11187128 | 10 | 94429708 | -0.0148 | 0.0025 | T | 4.78E-09 | 35.0464 |
| rs2419886 | 10 | 115841641 | -0.0192 | 0.0026 | T | 2.99E-13 | 54.53254438 |
| rs2762630 | 10 | 9265199 | 0.0317 | 0.0027 | G | 2.83E-31 | 137.8449931 |
| rs4935009 | 10 | 52829393 | -0.0193 | 0.003 | C | 2.33E-10 | 41.38777778 |
| rs11187838 | 10 | 96038686 | -0.018 | 0.0022 | A | 4.97E-16 | 66.94214876 |
| rs4938642 | 11 | 119099906 | 0.0287 | 0.0037 | C | 1.81E-14 | 60.16727538 |
| rs73632745 | 11 | 126229617 | -0.0611 | 0.0048 | T | 9.31E-38 | 162.031684 |
| rs2004315 | 11 | 13508384 | 0.0315 | 0.0023 | T | 1.93E-42 | 187.5708885 |
| rs12793417 | 11 | 34622050 | 0.0172 | 0.0026 | T | 8.03E-11 | 43.76331361 |
| rs3841466 | 11 | 77925543 | -0.0158 | 0.0027 | TG | 9.86E-09 | 34.2441701 |
| rs7108820 | 11 | 101999974 | 0.0143 | 0.0022 | C | 1.98E-10 | 42.25 |
| rs2583435 | 11 | 2958818 | -0.0233 | 0.0024 | C | 2.96E-22 | 94.25173611 |
| rs144562710 | 11 | 47933609 | 0.02 | 0.0026 | AAT | 1.37E-14 | 59.17159763 |
| rs4517550 | 11 | 71521071 | 0.0146 | 0.0023 | C | 3.91E-10 | 40.29489603 |
| rs302655 | 11 | 87891387 | -0.0186 | 0.0023 | T | 1.56E-16 | 65.39886578 |
| rs11228382 | 11 | 68617316 | -0.0182 | 0.0022 | T | 5.67E-16 | 68.43801653 |
| rs1182922 | 11 | 118932859 | -0.0134 | 0.0023 | A | 5.13E-09 | 33.94328922 |
| rs949300 | 11 | 122553139 | 0.0146 | 0.0023 | A | 1.20E-10 | 40.29489603 |
| rs775249 | 12 | 57977003 | -0.0155 | 0.0025 | T | 4.00E-10 | 38.44 |
| rs3026445 | 12 | 110723203 | -0.0172 | 0.0025 | C | 1.45E-11 | 47.3344 |
| rs17884869 | 12 | 123519112 | -0.1113 | 0.0079 | A | 1.05E-44 | 198.488864 |
| rs73202933 | 12 | 90213083 | 0.0235 | 0.0033 | C | 9.88E-13 | 50.71166208 |
| rs117213754 | 12 | 4006794 | 0.1036 | 0.0102 | A | 3.05E-24 | 103.1618608 |
| rs117080167 | 12 | 12205320 | -0.0318 | 0.0045 | T | 2.35E-12 | 49.93777778 |
| rs7964801 | 12 | 49097212 | -0.0271 | 0.0026 | T | 1.43E-24 | 108.6405325 |
| rs6580981 | 12 | 54723028 | -0.0152 | 0.0023 | A | 1.29E-11 | 43.67485822 |
| rs7323058 | 13 | 42548503 | -0.0348 | 0.0034 | T | 7.06E-24 | 104.7612457 |
| rs9524868 | 13 | 95909661 | -0.0135 | 0.0024 | T | 1.15E-08 | 31.640625 |
| rs2249825 | 13 | 31037903 | -0.0165 | 0.0026 | C | 2.55E-10 | 40.27366864 |
| rs1577452 | 13 | 110497532 | -0.0212 | 0.0025 | G | 4.88E-17 | 71.9104 |
| rs1023229 | 13 | 20270925 | -0.02 | 0.0034 | A | 7.21E-09 | 34.60207612 |
| rs35852840 | 14 | 64595763 | 0.029 | 0.0053 | A | 4.30E-08 | 29.93948024 |
| rs17580 | 14 | 94847262 | 0.0466 | 0.0058 | A | 5.51E-16 | 64.5529132 |
| rs58087925 | 14 | 105983096 | -0.0215 | 0.0029 | T | 2.83E-13 | 54.96432818 |
| rs28693943 | 14 | 60640916 | -0.0192 | 0.0029 | T | 7.66E-11 | 43.83353151 |
| rs28929474 | 14 | 94844947 | 0.1231 | 0.0088 | T | 2.20E-44 | 195.6819473 |
| rs11621792 | 14 | 24871926 | 0.0156 | 0.0024 | T | 1.54E-10 | 42.25 |
| rs7144433 | 14 | 90850229 | -0.0252 | 0.0033 | T | 4.76E-14 | 58.31404959 |
| rs11629876 | 15 | 96666402 | -0.0157 | 0.0024 | T | 8.21E-11 | 42.79340278 |
| rs4324076 | 15 | 51510868 | -0.0201 | 0.0022 | C | 9.14E-20 | 83.4731405 |
| rs60616569 | 15 | 78271261 | -0.0178 | 0.0028 | A | 1.70E-10 | 40.41326531 |
| rs2047824 | 15 | 69609456 | -0.0254 | 0.0023 | C | 5.25E-29 | 121.9584121 |
| rs139974673 | 15 | 44027885 | 0.1002 | 0.0079 | C | 1.58E-36 | 160.8722961 |
| rs41278174 | 16 | 16259596 | 0.0501 | 0.0076 | A | 4.26E-11 | 43.4558518 |
| rs34042070 | 16 | 72101525 | 0.0167 | 0.0027 | G | 3.80E-10 | 38.25651578 |
| rs12933858 | 16 | 81566121 | 0.0173 | 0.0023 | T | 5.55E-14 | 56.57655955 |
| rs73536752 | 16 | 47904346 | -0.0335 | 0.0061 | T | 4.14E-08 | 30.15990325 |
| rs12922549 | 16 | 54451747 | -0.0221 | 0.003 | T | 1.30E-13 | 54.26777778 |
| rs1858800 | 16 | 73024276 | 0.0266 | 0.0024 | T | 2.98E-28 | 122.8402778 |
| rs164751 | 16 | 89707636 | -0.0188 | 0.0025 | T | 1.26E-13 | 56.5504 |
| rs12918968 | 16 | 88520452 | -0.0331 | 0.0022 | C | 1.77E-50 | 226.3657025 |
| rs4790873 | 17 | 1994966 | -0.019 | 0.0025 | T | 4.94E-14 | 57.76 |
| rs6503506 | 17 | 37524653 | 0.0265 | 0.0026 | A | 1.08E-24 | 103.8831361 |
| rs12953299 | 17 | 17726648 | -0.0181 | 0.0024 | G | 1.19E-13 | 56.87673611 |
| rs228779 | 17 | 42091213 | -0.0181 | 0.0032 | G | 2.33E-08 | 31.99316406 |
| rs77542162 | 17 | 67081278 | -0.0894 | 0.0083 | G | 3.89E-27 | 116.0162578 |
| rs9895661 | 17 | 59456589 | -0.0256 | 0.0027 | T | 2.84E-21 | 89.89849108 |
| rs73342502 | 17 | 6612900 | 0.0329 | 0.0052 | C | 3.05E-10 | 40.02995562 |
| rs755736 | 17 | 47891904 | 0.0165 | 0.0026 | G | 2.10E-10 | 40.27366864 |
| rs11078597 | 17 | 1618363 | 0.0446 | 0.0028 | C | 2.93E-57 | 253.7193878 |
| rs35331358 | 18 | 60203140 | 0.0179 | 0.0027 | A | 7.25E-11 | 43.95198903 |
| rs73001065 | 19 | 19460541 | 0.0349 | 0.0048 | C | 4.20E-13 | 52.86501736 |
| rs1672991 | 19 | 35556659 | 0.0666 | 0.0043 | G | 1.82E-53 | 239.8896701 |
| rs75702986 | 19 | 35566151 | 0.0361 | 0.0031 | A | 1.33E-30 | 135.6097815 |
| rs117080418 | 19 | 50025208 | -0.099 | 0.0126 | A | 4.87E-15 | 61.73469388 |
| rs117896857 | 19 | 3111094 | -0.0553 | 0.0076 | T | 3.53E-13 | 52.94477147 |
| rs11881404 | 19 | 36021193 | -0.0176 | 0.0023 | G | 2.62E-14 | 58.5557656 |
| rs11085015 | 19 | 3369572 | -0.0181 | 0.0032 | G | 2.11E-08 | 31.99316406 |
| rs12982234 | 19 | 38586434 | -0.0582 | 0.0064 | T | 7.28E-20 | 82.69628906 |
| rs149807892 | 19 | 50159756 | 0.0659 | 0.01 | T | 5.38E-11 | 43.4281 |
| rs34944502 | 19 | 3121510 | 0.0427 | 0.0027 | GC | 1.83E-54 | 250.1083676 |
| rs2335534 | 19 | 50014977 | -0.0358 | 0.0031 | A | 5.40E-30 | 133.3652445 |
| rs35118755 | 19 | 52172672 | 0.023 | 0.0035 | T | 4.86E-11 | 43.18367347 |
| rs3091842 | 20 | 39344272 | 0.094 | 0.0062 | A | 1.85E-51 | 229.8647242 |
| rs17216707 | 20 | 52732362 | -0.0581 | 0.003 | C | 3.83E-82 | 375.0677778 |
| rs11086449 | 20 | 52713520 | -0.017 | 0.003 | AG | 1.77E-08 | 32.11111111 |
| rs6073257 | 20 | 42561422 | 0.0133 | 0.0023 | T | 7.24E-09 | 33.43856333 |
| rs11546155 | 20 | 33451148 | -0.0174 | 0.0031 | A | 2.35E-08 | 31.50468262 |
| rs73075609 | 20 | 5580789 | 0.056 | 0.0076 | T | 2.42E-13 | 54.29362881 |
| rs2762943 | 20 | 52790786 | 0.0505 | 0.0046 | G | 1.56E-27 | 120.5222117 |
| rs928760 | 21 | 35890958 | -0.0181 | 0.0023 | T | 1.22E-14 | 61.93005671 |
| rs12626330 | 21 | 37835982 | -0.0129 | 0.0022 | G | 6.18E-09 | 34.3822314 |
| rs5997623 | 22 | 30756907 | 0.0147 | 0.0025 | A | 3.51E-09 | 34.5744 |
| rs2017188 | 22 | 24994708 | 0.0146 | 0.0023 | C | 3.87E-10 | 40.29489603 |
| rs143193015 | 22 | 43110751 | 0.0228 | 0.0038 | GA | 2.30E-09 | 36 |

Supplementary Table 4. 118 SNPs associated with Vitamin D.

| SNP | Chromosome | position | Beta | Se | Effect allele | P | F |
| --- | --- | --- | --- | --- | --- | --- | --- |
| rs11207969 | 1 | 62911751 | 0.0209396 | 0.00212672 | G | 7.14E-23 | 96.94292785 |
| rs11264361 | 1 | 155289545 | 0.0174875 | 0.00234081 | G | 7.97E-14 | 55.811427 |
| rs61747728 | 1 | 179526214 | 0.0303061 | 0.00526894 | T | 8.83E-09 | 33.08366371 |
| rs2807834 | 1 | 220970593 | -0.0150625 | 0.00218678 | G | 5.66E-12 | 47.44428798 |
| rs512083 | 1 | 46027355 | 0.0122172 | 0.00204286 | C | 2.23E-09 | 35.76565288 |
| rs6672758 | 1 | 230303512 | 0.0162478 | 0.0025554 | T | 2.04E-10 | 40.42698452 |
| rs2494429 | 1 | 2339395 | -0.0148459 | 0.00267333 | G | 2.80E-08 | 30.83954186 |
| rs1343776 | 1 | 41757718 | 0.0180762 | 0.00245028 | A | 1.62E-13 | 54.42304602 |
| rs7528419 | 1 | 109817192 | 0.0215389 | 0.00243165 | G | 8.17E-19 | 78.45938691 |
| rs115288876 | 1 | 152000117 | 0.0788065 | 0.00498229 | A | 2.36E-56 | 250.187769 |
| rs35823191 | 1 | 17560123 | -0.0232636 | 0.00214072 | C | 1.65E-27 | 118.0957071 |
| rs61813875 | 1 | 152536650 | 0.0821291 | 0.00658876 | G | 1.16E-35 | 155.3770067 |
| rs2398113 | 10 | 10076429 | -0.0117606 | 0.00205797 | G | 1.10E-08 | 32.65734536 |
| rs12775091 | 10 | 91524012 | 0.0155618 | 0.00247693 | T | 3.33E-10 | 39.47227814 |
| rs2297991 | 10 | 113913222 | 0.0127547 | 0.00225578 | C | 1.57E-08 | 31.97032216 |
| rs77532868 | 10 | 88081438 | 0.0259557 | 0.00456301 | T | 1.28E-08 | 32.35658291 |
| rs144965707 | 11 | 14059511 | -0.0348143 | 0.00421754 | A | 1.52E-16 | 68.1391818 |
| rs1627043 | 11 | 71110175 | -0.0486441 | 0.00566106 | C | 8.49E-18 | 73.83543357 |
| rs2847500 | 11 | 120114421 | -0.022548 | 0.00308665 | A | 2.77E-13 | 53.36312795 |
| rs17473257 | 11 | 14283186 | -0.0611372 | 0.00780044 | A | 4.59E-15 | 61.42892117 |
| rs117300835 | 11 | 15118975 | -0.334985 | 0.00886005 | A | 1.00E-200 | 1429.480962 |
| rs2511279 | 11 | 71130419 | 0.0981721 | 0.00520826 | G | 2.98E-79 | 355.2964346 |
| rs3829251 | 11 | 71194559 | -0.114453 | 0.00298056 | A | 1.00E-200 | 1474.547013 |
| rs11023159 | 11 | 14262063 | 0.0482117 | 0.00572507 | C | 3.73E-17 | 70.91584607 |
| rs733454 | 11 | 76477721 | 0.0188545 | 0.00340001 | T | 2.93E-08 | 30.75173695 |
| rs111515741 | 11 | 14370944 | -0.0487364 | 0.00779044 | A | 3.95E-10 | 39.13658418 |
| rs12283049 | 11 | 14690192 | -0.0564566 | 0.00240614 | G | 9.62E-122 | 550.5384478 |
| rs11600054 | 11 | 14690511 | 0.0681747 | 0.0101478 | A | 1.84E-11 | 45.13388028 |
| rs964184 | 11 | 116648917 | 0.0406845 | 0.00298936 | C | 3.50E-42 | 185.2258208 |
| rs61887421 | 11 | 70949673 | -0.036726 | 0.00597754 | C | 8.05E-10 | 37.74872416 |
| rs7955128 | 12 | 38684121 | 0.0130617 | 0.00203856 | T | 1.48E-10 | 41.05371024 |
| rs1038165 | 12 | 68665940 | 0.0115149 | 0.00205629 | T | 2.15E-08 | 31.35823529 |
| rs73413596 | 12 | 111582630 | 0.0223468 | 0.00388928 | C | 9.15E-09 | 33.01355277 |
| rs28435470 | 12 | 133067473 | -0.0118696 | 0.00214831 | A | 3.29E-08 | 30.52658814 |
| rs57601828 | 12 | 93192127 | 0.011542 | 0.00208192 | T | 2.96E-08 | 30.73506001 |
| rs1871395 | 12 | 21352315 | -0.0203733 | 0.00282689 | G | 5.72E-13 | 51.94035834 |
| rs2171427 | 12 | 24822154 | -0.0165489 | 0.00281738 | A | 4.26E-09 | 34.50224928 |
| rs10859995 | 12 | 96375682 | -0.0436264 | 0.00205452 | C | 4.60E-100 | 450.8976869 |
| rs4580037 | 13 | 55702646 | -0.0135627 | 0.00225079 | C | 1.68E-09 | 36.30967461 |
| rs8018720 | 14 | 39556185 | -0.0344962 | 0.00266074 | C | 1.94E-38 | 168.0883589 |
| rs2756119 | 14 | 104001517 | 0.0121434 | 0.0021104 | A | 8.71E-09 | 33.10937976 |
| rs142004400 | 14 | 50829560 | -0.0310034 | 0.00559561 | C | 3.01E-08 | 30.69896769 |
| rs1532085 | 15 | 58683366 | 0.0252805 | 0.00208639 | G | 8.60E-34 | 146.8183481 |
| rs1800588 | 15 | 58723675 | -0.0305021 | 0.00246932 | T | 4.73E-35 | 152.5825024 |
| rs62007299 | 15 | 77711719 | -0.0124205 | 0.00224413 | A | 3.12E-08 | 30.63247835 |
| rs325393 | 15 | 100229260 | -0.0136497 | 0.00227676 | T | 2.03E-09 | 35.94278352 |
| rs12324720 | 15 | 64092140 | -0.0149159 | 0.00267451 | A | 2.45E-08 | 31.10358681 |
| rs1684600 | 16 | 4594671 | -0.0125301 | 0.00221719 | T | 1.59E-08 | 31.93767166 |
| rs11542462 | 16 | 82033810 | -0.0247803 | 0.00298265 | A | 9.72E-17 | 69.02533638 |
| rs77924615 | 16 | 20392332 | -0.0152475 | 0.00259019 | A | 3.94E-09 | 34.65245748 |
| rs11076175 | 16 | 57006378 | 0.0229033 | 0.00266997 | G | 9.64E-18 | 73.58399458 |
| rs11867297 | 17 | 66433493 | 0.0135432 | 0.00209454 | T | 1.01E-10 | 41.80856551 |
| rs61698755 | 17 | 79257880 | -0.011465 | 0.0020505 | C | 2.25E-08 | 31.26285029 |
| rs9946771 | 18 | 28918628 | -0.0233992 | 0.00407655 | T | 9.47E-09 | 32.94704523 |
| rs2037511 | 18 | 61366207 | 0.0176624 | 0.00272732 | A | 9.41E-11 | 41.93988521 |
| rs77960347 | 18 | 47109955 | -0.0525688 | 0.00905963 | G | 6.53E-09 | 33.6693866 |
| rs10438978 | 18 | 47158186 | -0.0172243 | 0.00264437 | C | 7.34E-11 | 42.42664784 |
| rs1048328 | 19 | 51527364 | 0.0313497 | 0.00374376 | A | 5.58E-17 | 70.12143237 |
| rs142158911 | 19 | 11190534 | 0.026284 | 0.00323446 | A | 4.43E-16 | 66.03578543 |
| rs12462826 | 19 | 11955767 | -0.0132119 | 0.0021149 | A | 4.18E-10 | 39.02571614 |
| rs4420638 | 19 | 45422946 | -0.0192973 | 0.00265905 | G | 3.95E-13 | 52.66718295 |
| rs8107974 | 19 | 19388500 | 0.0355672 | 0.00382316 | T | 1.36E-20 | 86.54747593 |
| rs62129966 | 19 | 48374950 | 0.0611636 | 0.00276373 | A | 1.60E-108 | 489.7730152 |
| rs1042034 | 2 | 21225281 | -0.0151254 | 0.00250017 | T | 1.45E-09 | 36.59945833 |
| rs1260326 | 2 | 27730940 | 0.0197194 | 0.00207421 | C | 1.96E-21 | 90.38199967 |
| rs35270497 | 2 | 38259872 | 0.0156723 | 0.0026815 | T | 5.08E-09 | 34.15937139 |
| rs7569755 | 2 | 118648261 | 0.0136395 | 0.00225626 | A | 1.49E-09 | 36.54421288 |
| rs2710651 | 2 | 63166379 | -0.0115892 | 0.00203474 | A | 1.23E-08 | 32.44061434 |
| rs3732220 | 2 | 234627048 | -0.0478406 | 0.00363266 | A | 1.31E-39 | 173.4377904 |
| rs727857 | 2 | 58981967 | -0.0120548 | 0.00209882 | A | 9.27E-09 | 32.98903646 |
| rs7580771 | 2 | 101428119 | -0.0165625 | 0.0026652 | T | 5.15E-10 | 38.61821298 |
| rs1047891 | 2 | 211540507 | -0.0133984 | 0.00218016 | A | 7.96E-10 | 37.76844843 |
| rs11207969 | 1 | 62911751 | 0.0209396 | 0.00212672 | G | 7.14E-23 | 96.94292785 |
| rs11264361 | 1 | 155289545 | 0.0174875 | 0.00234081 | G | 7.97E-14 | 55.811427 |
| rs61747728 | 1 | 179526214 | 0.0303061 | 0.00526894 | T | 8.83E-09 | 33.08366371 |
| rs2807834 | 1 | 220970593 | -0.0150625 | 0.00218678 | G | 5.66E-12 | 47.44428798 |
| rs512083 | 1 | 46027355 | 0.0122172 | 0.00204286 | C | 2.23E-09 | 35.76565288 |
| rs6672758 | 1 | 230303512 | 0.0162478 | 0.0025554 | T | 2.04E-10 | 40.42698452 |
| rs2494429 | 1 | 2339395 | -0.0148459 | 0.00267333 | G | 2.80E-08 | 30.83954186 |
| rs1343776 | 1 | 41757718 | 0.0180762 | 0.00245028 | A | 1.62E-13 | 54.42304602 |
| rs7528419 | 1 | 109817192 | 0.0215389 | 0.00243165 | G | 8.17E-19 | 78.45938691 |
| rs115288876 | 1 | 152000117 | 0.0788065 | 0.00498229 | A | 2.36E-56 | 250.187769 |
| rs35823191 | 1 | 17560123 | -0.0232636 | 0.00214072 | C | 1.65E-27 | 118.0957071 |
| rs61813875 | 1 | 152536650 | 0.0821291 | 0.00658876 | G | 1.16E-35 | 155.3770067 |
| rs2398113 | 10 | 10076429 | -0.0117606 | 0.00205797 | G | 1.10E-08 | 32.65734536 |
| rs12775091 | 10 | 91524012 | 0.0155618 | 0.00247693 | T | 3.33E-10 | 39.47227814 |
| rs2297991 | 10 | 113913222 | 0.0127547 | 0.00225578 | C | 1.57E-08 | 31.97032216 |
| rs77532868 | 10 | 88081438 | 0.0259557 | 0.00456301 | T | 1.28E-08 | 32.35658291 |
| rs144965707 | 11 | 14059511 | -0.0348143 | 0.00421754 | A | 1.52E-16 | 68.1391818 |
| rs1627043 | 11 | 71110175 | -0.0486441 | 0.00566106 | C | 8.49E-18 | 73.83543357 |
| rs2847500 | 11 | 120114421 | -0.022548 | 0.00308665 | A | 2.77E-13 | 53.36312795 |
| rs17473257 | 11 | 14283186 | -0.0611372 | 0.00780044 | A | 4.59E-15 | 61.42892117 |
| rs117300835 | 11 | 15118975 | -0.334985 | 0.00886005 | A | 1.00E-200 | 1429.480962 |
| rs2511279 | 11 | 71130419 | 0.0981721 | 0.00520826 | G | 2.98E-79 | 355.2964346 |
| rs3829251 | 11 | 71194559 | -0.114453 | 0.00298056 | A | 1.00E-200 | 1474.547013 |
| rs11023159 | 11 | 14262063 | 0.0482117 | 0.00572507 | C | 3.73E-17 | 70.91584607 |
| rs733454 | 11 | 76477721 | 0.0188545 | 0.00340001 | T | 2.93E-08 | 30.75173695 |
| rs111515741 | 11 | 14370944 | -0.0487364 | 0.00779044 | A | 3.95E-10 | 39.13658418 |
| rs12283049 | 11 | 14690192 | -0.0564566 | 0.00240614 | G | 9.62E-122 | 550.5384478 |
| rs11600054 | 11 | 14690511 | 0.0681747 | 0.0101478 | A | 1.84E-11 | 45.13388028 |
| rs964184 | 11 | 116648917 | 0.0406845 | 0.00298936 | C | 3.50E-42 | 185.2258208 |
| rs61887421 | 11 | 70949673 | -0.036726 | 0.00597754 | C | 8.05E-10 | 37.74872416 |
| rs7955128 | 12 | 38684121 | 0.0130617 | 0.00203856 | T | 1.48E-10 | 41.05371024 |
| rs1038165 | 12 | 68665940 | 0.0115149 | 0.00205629 | T | 2.15E-08 | 31.35823529 |
| rs73413596 | 12 | 111582630 | 0.0223468 | 0.00388928 | C | 9.15E-09 | 33.01355277 |
| rs28435470 | 12 | 133067473 | -0.0118696 | 0.00214831 | A | 3.29E-08 | 30.52658814 |
| rs57601828 | 12 | 93192127 | 0.011542 | 0.00208192 | T | 2.96E-08 | 30.73506001 |
| rs1871395 | 12 | 21352315 | -0.0203733 | 0.00282689 | G | 5.72E-13 | 51.94035834 |
| rs2171427 | 12 | 24822154 | -0.0165489 | 0.00281738 | A | 4.26E-09 | 34.50224928 |
| rs10859995 | 12 | 96375682 | -0.0436264 | 0.00205452 | C | 4.60E-100 | 450.8976869 |
| rs4580037 | 13 | 55702646 | -0.0135627 | 0.00225079 | C | 1.68E-09 | 36.30967461 |
| rs8018720 | 14 | 39556185 | -0.0344962 | 0.00266074 | C | 1.94E-38 | 168.0883589 |
| rs2756119 | 14 | 104001517 | 0.0121434 | 0.0021104 | A | 8.71E-09 | 33.10937976 |
| rs142004400 | 14 | 50829560 | -0.0310034 | 0.00559561 | C | 3.01E-08 | 30.69896769 |
| rs1532085 | 15 | 58683366 | 0.0252805 | 0.00208639 | G | 8.60E-34 | 146.8183481 |
| rs1800588 | 15 | 58723675 | -0.0305021 | 0.00246932 | T | 4.73E-35 | 152.5825024 |
| rs62007299 | 15 | 77711719 | -0.0124205 | 0.00224413 | A | 3.12E-08 | 30.63247835 |
| rs325393 | 15 | 100229260 | -0.0136497 | 0.00227676 | T | 2.03E-09 | 35.94278352 |

Supplementary Table 5. 68 SNPs associated with Vitamin C.

| SNP | Chromosome | position | Beta | Se | Effect allele | P | F |
| --- | --- | --- | --- | --- | --- | --- | --- |
| rs147412044 | 1 | 167758303 | -0.8188 | 0.1311 | T | 4.26E-10 | 39.00769468 |
| rs115757213 | 1 | 211685549 | -0.3982 | 0.0725 | G | 3.93E-08 | 30.16660927 |
| rs145396741 | 1 | 225599808 | -0.3569 | 0.0611 | A | 5.20E-09 | 34.12012986 |
| rs77503249 | 1 | 234790766 | -0.3558 | 0.052 | A | 7.92E-12 | 46.81717456 |
| rs35248101 | 10 | 13171617 | -0.2336 | 0.0409 | C | 1.16E-08 | 32.6211345 |
| rs138662264 | 10 | 101464483 | -0.8716 | 0.1062 | C | 2.31E-16 | 67.35741468 |
| rs141694582 | 10 | 30833134 | -0.3648 | 0.0649 | A | 1.93E-08 | 31.59513866 |
| rs77992796 | 10 | 129203425 | -0.4948 | 0.0831 | A | 2.66E-09 | 35.45335459 |
| rs3026057 | 10 | 119014127 | -0.8949 | 0.1352 | A | 3.60E-11 | 43.81225765 |
| rs118027537 | 12 | 16085254 | -0.8831 | 0.1303 | C | 1.22E-11 | 45.93364801 |
| rs77459185 | 12 | 77206531 | -0.3061 | 0.0454 | G | 1.54E-11 | 45.45848454 |
| rs77820382 | 12 | 111511225 | -0.5747 | 0.0767 | A | 6.84E-14 | 56.14248949 |
| rs145119123 | 13 | 108479750 | -0.2923 | 0.0526 | T | 2.81E-08 | 30.88062933 |
| rs117042417 | 13 | 66201226 | -0.4897 | 0.0789 | A | 5.34E-10 | 38.52176714 |
| rs142070515 | 13 | 92501248 | -0.6196 | 0.1003 | G | 6.49E-10 | 38.16110591 |
| rs146783469 | 14 | 60859899 | -0.7732 | 0.1311 | G | 3.64E-09 | 34.78390268 |
| rs143302471 | 16 | 2895094 | -0.5206 | 0.086 | A | 1.39E-09 | 36.64472147 |
| rs141568393 | 16 | 22944021 | -0.4512 | 0.0805 | G | 2.08E-08 | 31.41567686 |
| rs373008011 | 16 | 84127498 | -0.5831 | 0.0904 | T | 1.09E-10 | 41.60535403 |
| rs17637408 | 16 | 26295044 | -0.2981 | 0.0527 | C | 1.49E-08 | 31.99651819 |
| rs1399998 | 16 | 27163332 | -0.753 | 0.1307 | C | 8.36E-09 | 33.1924093 |
| rs117193023 | 18 | 5643874 | -0.355 | 0.0636 | T | 2.35E-08 | 31.15605593 |
| rs80148899 | 18 | 22668598 | -0.9042 | 0.1051 | T | 7.71E-18 | 74.01565271 |
| rs12454633 | 18 | 40494893 | -0.3071 | 0.0532 | A | 7.89E-09 | 33.32240729 |
| rs12462583 | 19 | 38905792 | -0.2106 | 0.0358 | A | 4.24E-09 | 34.60594239 |
| rs116716302 | 2 | 140681452 | -0.461 | 0.0784 | T | 4.15E-09 | 34.57560001 |
| rs146985384 | 2 | 141602409 | -0.7361 | 0.1332 | G | 3.25E-08 | 30.53972948 |
| rs147589962 | 2 | 185869804 | -0.426 | 0.0706 | C | 1.62E-09 | 36.40908763 |
| rs114637089 | 2 | 48801442 | -0.3821 | 0.0696 | A | 4.03E-08 | 30.13946897 |
| rs73995784 | 2 | 235971015 | -0.4812 | 0.0785 | G | 8.70E-10 | 37.57611911 |
| rs150674662 | 2 | 122911880 | -0.3976 | 0.0704 | A | 1.60E-08 | 31.89682335 |
| rs6030549 | 20 | 41519220 | -0.645 | 0.1114 | C | 7.00E-09 | 33.5234763 |
| rs187748425 | 21 | 40558118 | -0.5082 | 0.071 | A | 8.40E-13 | 51.23333466 |
| rs147668949 | 21 | 28314693 | -0.7291 | 0.131 | A | 2.61E-08 | 30.97644718 |
| rs149503879 | 21 | 45271053 | -0.5354 | 0.0866 | A | 6.31E-10 | 38.22266373 |
| rs113757848 | 22 | 34545881 | -0.538 | 0.0897 | A | 2.01E-09 | 35.97324912 |
| rs12490747 | 3 | 9092890 | -0.2677 | 0.0484 | A | 3.19E-08 | 30.59186958 |
| rs71323476 | 3 | 116616727 | -0.6054 | 0.1104 | T | 4.13E-08 | 30.07091801 |
| rs116768603 | 3 | 1348020 | -0.3479 | 0.0507 | G | 7.14E-12 | 47.08612366 |
| rs4859019 | 3 | 88440502 | -0.4399 | 0.0701 | C | 3.44E-10 | 39.37965328 |
| rs115504365 | 3 | 136506946 | -0.5922 | 0.1041 | A | 1.26E-08 | 32.36199952 |
| rs2222837 | 3 | 160331821 | -0.4193 | 0.0769 | A | 4.88E-08 | 29.73014622 |
| rs115036356 | 3 | 13191877 | -0.4144 | 0.0744 | A | 2.55E-08 | 31.02370216 |
| rs149277200 | 3 | 184405033 | -0.5132 | 0.0861 | G | 2.53E-09 | 35.52769178 |
| rs141109644 | 3 | 196034184 | -0.3335 | 0.0604 | G | 3.39E-08 | 30.48721821 |
| rs73122527 | 4 | 27134001 | -0.5183 | 0.0863 | A | 1.94E-09 | 36.06955848 |
| rs151079977 | 4 | 95995355 | -0.3436 | 0.0593 | G | 6.76E-09 | 33.5735236 |
| rs116058454 | 4 | 82799684 | -0.7361 | 0.1332 | A | 3.25E-08 | 30.53972948 |
| rs147393151 | 4 | 138234060 | -0.5488 | 0.0938 | T | 4.91E-09 | 34.2312319 |
| rs202139829 | 4 | 144621585 | -0.2837 | 0.052 | A | 4.90E-08 | 29.7654179 |
| rs62389352 | 5 | 163304133 | -0.6152 | 0.0682 | T | 1.95E-19 | 81.36992286 |
| rs111266167 | 5 | 132512946 | -0.4258 | 0.0761 | T | 2.25E-08 | 31.30703946 |
| rs145489384 | 5 | 92700914 | -0.2956 | 0.0523 | G | 1.59E-08 | 31.94519046 |
| rs116106286 | 5 | 172207791 | -0.4276 | 0.0727 | A | 4.08E-09 | 34.59446123 |
| rs6911990 | 6 | 152669178 | -0.4612 | 0.0768 | C | 1.92E-09 | 36.06252713 |
| rs16889083 | 6 | 36729068 | -0.2534 | 0.0396 | T | 1.52E-10 | 40.94707173 |
| rs239825 | 6 | 54773200 | -0.4749 | 0.08 | C | 2.95E-09 | 35.23906406 |
| rs146857350 | 6 | 112518469 | -0.5956 | 0.079 | A | 4.77E-14 | 56.84014741 |
| rs116967294 | 6 | 95056239 | -0.7594 | 0.1334 | C | 1.26E-08 | 32.40630584 |
| rs76715584 | 6 | 152513466 | -0.2278 | 0.0379 | T | 1.86E-09 | 36.12676047 |
| rs144580260 | 7 | 48052891 | -0.519 | 0.0859 | A | 1.54E-09 | 36.50466674 |
| rs143142132 | 7 | 12082686 | -0.8303 | 0.1305 | T | 1.96E-10 | 40.48079682 |
| rs181246816 | 8 | 1278957 | -0.2302 | 0.0398 | T | 7.38E-09 | 33.45372592 |
| rs77222063 | 8 | 21773577 | -0.8769 | 0.1134 | G | 1.02E-14 | 59.7962613 |
| rs35664127 | 9 | 73622580 | -0.411 | 0.0702 | A | 4.90E-09 | 34.2775221 |
| rs139991506 | 9 | 23704505 | -0.8069 | 0.1311 | G | 7.42E-10 | 37.88210012 |
| rs190279780 | 9 | 8949161 | -0.416 | 0.0687 | A | 1.44E-09 | 36.66681498 |
| rs116559716 | 9 | 98210170 | -0.5459 | 0.0968 | A | 1.69E-08 | 31.80354078 |

Supplementary Table 6. 2 SNPs associated with Zinc.

| SNP | Chromosome | position | Beta | Se | Effect allele | P | F |
| --- | --- | --- | --- | --- | --- | --- | --- |
| rs1532423 | 8 | 86268313 | -0.178 | 0.026 | G | 6.40E-12 | 46.86982249 |
| rs2120019 | 15 | 75334184 | -0.287 | 0.033 | C | 1.55E-18 | 75.63728191 |
